# Hydrothermal synthesis of ZnZrO_*x*_ catalysts for CO_2_ hydrogenation to methanol: the effect of pH on structure and activity†

**DOI:** 10.1039/d4su00522h

**Published:** 2024-10-11

**Authors:** Issaraporn Rakngam, Gustavo A. S. Alves, Nattawut Osakoo, Jatuporn Wittayakun, Thomas Konegger, Karin Föttinger

**Affiliations:** a School of Chemistry, Institute of Science, Suranaree University of Technology Nakhon Ratchasima 30000 Thailand; b Institute of Materials Chemistry, TU Wien Getreidemarkt 9 1060 Vienna Austria karin.foettinger@tuwien.ac.at; c Chair of Physical Chemistry, Montanuniversität Leoben Franz-Josef-Straße 18 8700 Leoben Austria; d Institute of Research and Development, Suranaree University of Technology Thailand; e Institute of Chemical Technologies and Analytics, TU Wien Getreidemarkt 9 1060 Vienna Austria

## Abstract

With the growing necessity of achieving carbon neutrality in the industrial sector, the catalytic hydrogenation of carbon dioxide into methanol has been widely considered one of the key strategies for the utilization of captured CO_2_. For this reason, the development of alternative catalysts such as ZnZrO_*x*_ has attracted considerable interest, given its superior stability and versatility in comparison to the conventional Cu-based materials. In this work, ZnZrO_*x*_ has been produced by a hydrothermal synthesis method at varied synthesis pH between 7 and 10 and a positive association between pH and catalytic CO_2_ conversion is observed. At 2.0 MPa and 250 °C, ZnZrO_*x*_ produced at pH 10 shows a methanol selectivity of 95% at a CO_2_ conversion of 3.4%. According to characterization, basic pH conditions enable the formation of abundant t-ZrO_2_ and the subsequent incorporation of Zn^2+^ into this phase, although the content of surface Zn does not increase between pH 8 and 10. Nevertheless, synthesis pH values can be correlated with surface oxygen content and CO_2_ adsorption capacity, which could be important contributors to the higher catalytic activity observed as a result of higher synthesis pH values. However, upon synthesis at pH 10, an inferior selectivity to methanol is observed above 250 °C, as a possible result of the excessive formation of ZnO. Interestingly, this secondary phase can be prevented and the selectivity can be slightly improved by utilizing NH_4_OH instead of NaOH in the hydrothermal method.

## Abstract 2

With the growing necessity of achieving carbon neutrality in the industrial sector, the catalytic hydrogenation of carbon dioxide into methanol has been widely considered one of the key strategies for the utilization of captured CO_2_.

## Graphical abstract

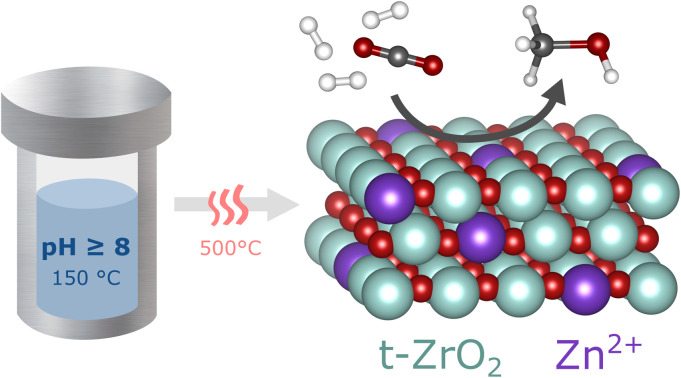

**Sustainability spotlight**

One of the key building blocks in the chemical industry, methanol, has been primarily obtained from fossil feedstocks over the last decades. With the growing necessity of developing sustainable alternatives, the production of renewable methanol from CO_2_ has been recently proposed as a strategy to utilize carbon derived from biomass or industrial emissions from hard-to-abate sectors. In this context, the development of more stable and versatile catalysts may facilitate the implementation of CO_2_ hydrogenation to methanol on a large scale. This research work addresses the following Sustainable Development Goals: Industry, Innovation and Infrastructure (SDG 9), Sustainable Consumption and Production (SDG 12) and Climate Action (SDG 13).

## Introduction

The relentless increase in atmospheric carbon dioxide (CO_2_) levels and the urgent need to mitigate climate change have been motivating intensive research into sustainable solutions for CO_2_ utilization,^1^ often involving its hydrogenation into carbon monoxide,^2^ alcohols^3^ and olefins.^4^ Among these strategies, the conversion of CO_2_ to methanol has been considered a promising route for the utilization of CO_2_ emissions due to the versatility and energy density of methanol as a liquid fuel and chemical feedstock. Typically, the industrial synthesis of methanol from CO-rich syngas at high pressure employs the Cu/ZnO/Al_2_O_3_ catalyst, which can also be applied for the direct hydrogenation of CO_2_.^5^ Nevertheless, one of the drawbacks of such copper-based materials is the deactivation due to the limited stability of the catalyst under exposure to moisture and sulfur-containing gases, which may pose a considerable obstacle to the continuous long-term operation of the methanol synthesis from industrial CO_2_ feedstocks.^6,7^ For this reason, the development of more stable and robust catalysts could benefit the yet incipient production of renewable methanol derived from a variety of CO_2_ sources, such as biogas,^8^ geothermal origins^9^ and steel plants.^10^

Among the diverse catalysts explored for CO_2_ hydrogenation to methanol, ZnZrO_*x*_-based materials have demonstrated notable potential as next-generation catalysts, given their excellent selectivity, stability and sulfur tolerance.^11,12^ On the other hand, their non-metallic character engenders limited hydrogen activation, leading to lower CO_2_ conversion compared to commercial Cu/ZnO-based materials.^13^

In ZnZrO_*x*_ catalysts, the synergistic interaction between Zn^2+^ and ZrO_2_ plays a vital role in promoting both catalytic activity and selectivity.^11^ Specifically, the Zn^2+^–O–Zr^4+^ sites from the ZnZrO_*x*_ solid solution are identified as the active sites for CO_2_-to-methanol hydrogenation,^14,15^ in order that Zn species are considered responsible for dissociating H_2_ molecules, while Zr species facilitate the activation of CO_2_.^15^ This catalytically active Zn^2+^–O–Zr^4+^ system has been mostly observed as a result of Zn^2+^ ions incorporated in the tetragonal ZrO_2_ lattice (t-ZrO_2_), while the monoclinic zirconia polymorph (m-ZrO_2_) is considered less able to accommodate these species.^16^ Additionally, the recent evidence for ZnO clusters embedded in ZrO_2_ as a key feature of ZnZrO_*x*_ catalysts indicates that ZnO/ZrO_2_ systems should be also taken into account as possible active sites for CO_2_ hydrogenation in these materials.^17,18^ In addition to the clear importance of Zn^2+^ species in ZnZrO_*x*_, recent studies have emphasized the role of lattice oxygen on H_2_ activation, suggesting a direct correlation between catalytic activity and surface oxygen content.^19^ In fact, experimental and computational studies indicate that Zn^2+^–O^2−^ pairs may be responsible for the heterolytic H_2_ dissociation in ZnZrO_*x*_ catalysts for CO_2_ hydrogenation to methanol.^20^

In several previous studies, ZnZrO_*x*_ catalysts with a high content of t-ZrO_2_ have been typically produced by co-precipitation approaches, followed by calcination at 500 °C.^11,21,22^ Although t-ZrO_2_ is thermodynamically less stable than m-ZrO_2_ at such temperatures, the presence of a hydrated surface^23^ and small crystallites^24^ may promote the formation of tetragonal zirconia in these cases. Alternatively, a hydrothermal approach followed by calcination between 300 °C and 600 °C has been shown as an effective method to produce t-ZrO_2_-based catalysts, and the presence of Na ions has been suggested as another important factor for the stabilization of the tetragonal phase.^25^ Therefore, the hydrothermal synthesis method may deserve further exploration due to the possibility of obtaining nanostructured ZnZrO_*x*_ catalysts with high surface area^26^ and suitable crystalline structure for CO_2_ hydrogenation to methanol. However, achieving optimal catalytic performance requires a deeper understanding of the catalyst synthesis under varying parameters, such as pH values, which are often a key factor in the nucleation, growth, and crystal size of metal-oxide particles. In Zn and Zr aqueous solutions, basic pH was shown to accelerate the crystallization of Zn and Zr oxides.^27,28^ Thus, exploring the catalytic improvement of ZnZrO_*x*_ by systematically varying pH levels represents a promising strategy for enhancing the efficiency of CO_2_ conversion to methanol in this catalyst.

This work presents an investigation into the catalytic performance and material properties of ZnZrO_*x*_ produced by hydrothermal synthesis. Herein, the effect of synthesis pH on structural and surface properties is investigated and the material is tested as a catalyst for CO_2_ hydrogenation to methanol.

## Experimental

### Chemicals and materials

Zirconyl chloride octahydrate (ZrOCl_2_·8H_2_O, 98%, Sigma-Aldrich), zinc chloride (ZnCl_2_, 98%, Fluka), sodium hydroxide (NaOH, 98%, Sigma-Aldrich), and ammonium hydroxide (NH_4_OH, 25%, Donau Chem) were used for the catalyst synthesis.

### Synthesis of ZnZrO_*x*_

A series of ZnZrO_*x*_ catalysts were prepared at different pH values (7, 8, 9, and 10) using a hydrothermal treatment method based on a previously reported procedure for the preparation of t-ZrO_2_.^25^ Initially, 3.36 g of ZrOCl_2_·8H_2_O and ZnCl_2_ with the Zn/(Zr + Zn) mole ratio of 20% were dissolved in 20 mL of deionized water under stirring. Subsequently, a 1.0 M NaOH solution was slowly added into the mixed metal solution to achieve the desired pH value. The mixture was then transferred into an autoclave with a Teflon liner and heated at 150 °C for 18 h. After the hydrothermal treatment, the autoclave was cooled down to room temperature. The white powder was filtered, washed with deionized water until neutral pH, dried, and calcined at 500 °C for 3 h. Alternatively, a similar procedure was followed for the production of ZnZrO_*x*_ at pH 10, using NH_4_OH as a pH adjuster instead of NaOH.

### Catalyst characterization

The crystalline structure of the samples was characterized by powder X-ray diffraction (XRD) on a Philips XPert diffractometer using Cu Kα radiation (*λ* = 1.5406 Å) at 45 kV and 40 mA operating in Bragg–Brentano reflection geometry. Transmission Electron Microscopy (TEM) and Energy-Dispersive X-ray Spectroscopy (EDX) were performed on a Thermo Scientific TALOS F200X operated at 200 kV. The morphologies of the samples were investigated by scanning electron microscopy (SEM) with a FEI Quanta 250 FEG microscope at a 5 kV voltage. N_2_ adsorption–desorption analysis of the samples was determined using Micromeritics ASAP 2020 at −196 °C. Before the measurement, the sample was degassed at 350 °C for 8 h under vacuum. Specific surface areas were calculated using the Brunauer–Emmett–Teller (BET) method. Pore size distributions were determined by the Barrett–Joyner–Halenda (BJH) model.

The basicity and CO_2_ adsorption capacity of the sample were investigated by temperature-programmed desorption of carbon dioxide (CO_2_-TPD) using a BELCAT-B chemisorption analyzer with a thermal conductivity detector (TCD). Prior to analysis, the sample was pretreated at 350 °C for 1 h under flowing He gas with 30 mL min^−1^. Then, the sample was cooled down to 50 °C and a gas mixture containing 10% CO_2_ in He was adsorbed on the sample surface for 1 h. The sample was purged with He and held for 30 min to remove non-adsorbed CO_2_. The TPD process was performed in the temperature range from 50 to 350 °C with a heating rate of 10 °C min^−1^ and held at 350 °C for 1 h under a He flow of 30 mL min^−1^.

Chemical states of surface species were identified by X-ray photoelectron spectroscopy (XPS) with a SPECS u-Focus system (AlKα source, Phoibos 150 WAL detector). XPS data evaluation was carried out using the CasaXPS software,^29^ considering spectra calibrated with the C 1s peak at 284.8 eV. Quantification of surface species was conducted by considering the areas of Zn 2p_3/2_, O 1s, Zr 3p_3/2_, Zr 3d and Zn 3p peaks with the respective Relative Sensitivity Factors (RSF) of 18.92, 2.93, 5.14, 7.04 and 2.83.

*In situ* diffuse reflectance infrared Fourier transform spectroscopy (DRIFTS) was carried out on a Bruker Vertex 70 spectrometer equipped with a mercury-cadmium-telluride detector cooled by liquid nitrogen. Before measurement, the sample was pretreated at 350 °C for 2 h under pure H_2_ flow (7.7 mL min^−1^) and then cooled to 250 °C under Ar flow (11.5 mL min^−1^). After cooling down, the background spectrum was collected from 800–4000 cm^−1^ with 256 scans at a resolution of 4 cm^−1^. H_2_ (7.7 mL min^−1^) and CO_2_ (2.6 mL min^−1^) were introduced into the reaction cell and the spectra were collected at 250 °C.

### Catalytic testing for CO_2_ hydrogenation

The catalytic activity testing was performed in a tubular fixed-bed continuous-flow “micro effi” reactor from PID Eng&Tech. Prior to the test, 1 g of the ZnZrO_*x*_ catalyst was activated under H_2_ flow at 350 °C for 2 h. After cooling to 250 °C, the reactant gas mixture of CO_2_/H_2_/He (20/60/20) was introduced into the reactor at a total flow of 5 mL_n_/min under a pressure of 2.0 MPa. The reaction was conducted in steps of 10 °C between 250 to 290 °C during 6 h in each step. Detection of products in gas phase was carried out using an Inficon Micro GC 3000 equipped with a Plot Q column. The CO_2_ conversion (*X*) and the selectivity (*S*) for CH_3_OH, CO and CH_4_ were calculated using the following equations:1
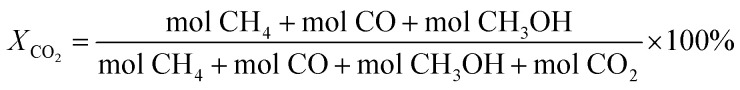
2
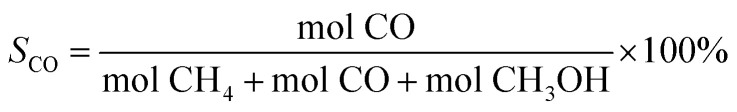
3
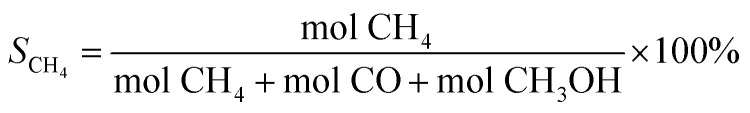
4
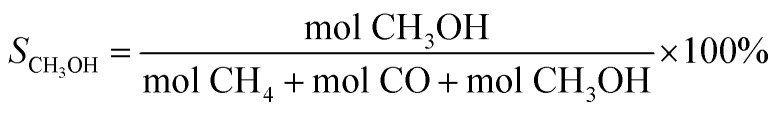


## Results and discussion

**Fig. 1:**
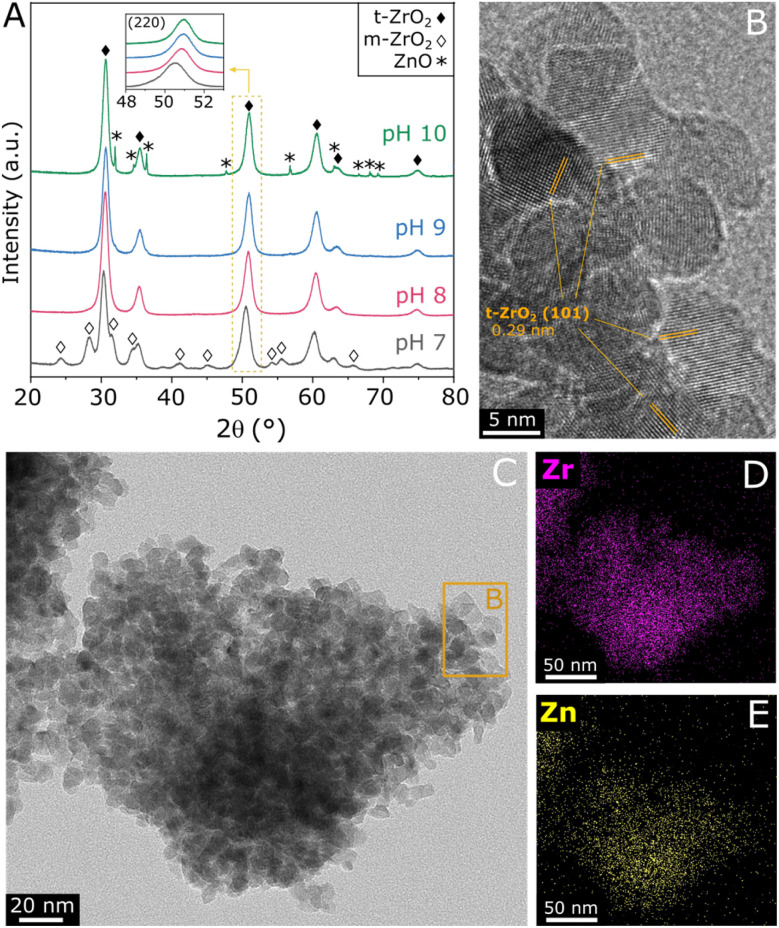
XRD patterns of ZnZrO_*x*_ produced at different synthesis pH values and calcined at 500 °C (A), TEM image of ZnZrO_*x*_ produced at pH 8 (B and C) and TEM-EDX mapping images showing the Zr and Zn distribution in the same region covered by C (D andE).

ZnZrO_*x*_ samples were produced by hydrothermal synthesis using NaOH to adjust pH values between 7 and 10. Considering that the saturation of t-ZrO_2_ with Zn^2+^ has been previously reported at 25%,^16^ a Zn/(Zn + Zr) atomic ratio of 20% was chosen, seeking to obtain abundant and dispersed Zn^2+^ sites in a ZnZrO_*x*_ without the formation of segregated ZnO. In order to verify the crystalline structure of the material, XRD analysis was conducted, as shown in Fig. 1A. The XRD pattern of ZnZrO_*x*_ prepared at pH 7 presents evidence for the tetragonal ZrO_2_ (t-ZrO_2_) phase (JCPDS 68-0200) mixed with a monoclinic (m-ZrO_2_) phase (JCPDS 65-0687).^11^ However, more basic pH conditions completely attenuate the pattern related to m-ZrO_2_, as observed for the pH 8 and pH 9 samples. Upon increasing pH during synthesis to 10, additional small features emerge at 31.9°, 34.6°, 36.4°, 47.8°, 56.8°, 63.0°, and 68.1°, which can be assigned to hexagonal ZnO phase (JCPDS 01-083-6338). This result indicates that the material produced at pH 10 presents both the t-ZrO_2_ phase and a secondary contribution of hexagonal ZnO. Nevertheless, in all cases the material consists primarily of tetragonal zirconia, which is observed in more detail by TEM in Fig. 1B and C, showing the characteristic (101) interplanar spacing of 0.29 nm from t-ZrO_2_. As shown by TEM-EDS analysis in Fig. 1D and E, this phase presents a homogeneous distribution of Zr and Zn, giving evidence for the incorporation of Zn atoms into the t-ZrO_2_ structure. Furthermore, as shown in the insert of Fig. 1A, the diffraction feature corresponding to t-ZrO_2_ shifts by approximately 0.5° towards higher angles as the pH during synthesis increases from 7 to 9, but no changes are verified between pH 9 and 10. The observed shift can be attributed to the incorporation of Zn^2+^ ions (ionic radius 0.74 Å) into the t-ZrO_2_ lattice, leading to a reduction in interplanar spacing due to the substitution of Zr^4+^ (ionic radius, 0.82 Å) by smaller radius size of Zn^2+^ ions.^11,30^ Therefore, these findings indicate that basic hydrothermal conditions facilitate the formation of the Zn–ZrO_2_ solid solution. However, above pH 9 this phase may be already saturated with Zn^2+^, which leads to the formation of segregated ZnO crystallites at higher pH.

**Fig. 2:**
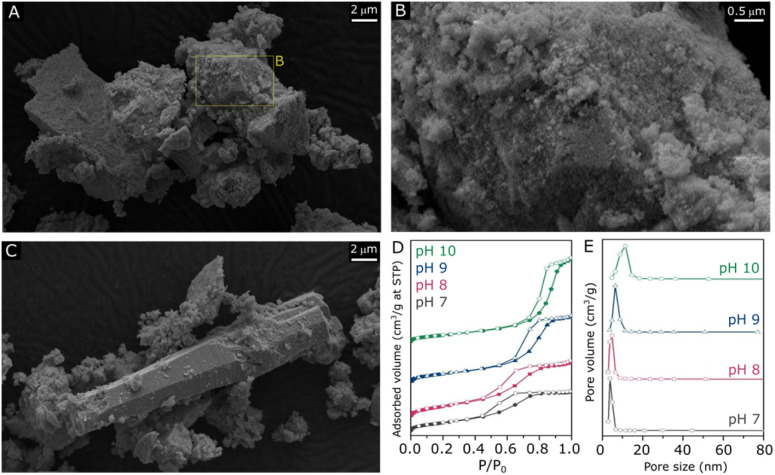
SEM micrographs of ZnZrO_*x*_ produced at pH10 under different magnifications (A and B) and the region including rod-like particles (C). N_2_ sorption isotherms (D) with respective pore size distributions of ZnZrO_*x*_ produced at pH 7, 8, 9 and 10 (E).

Following the verification of clear influences of synthesis pH on the crystalline structure of ZnZrO_*x*_, surface properties of the material have been evaluated by SEM, N_2_ physisorption, XPS and CO_2_-TPD. According to the SEM micrographs shown in Fig. 2 and S1–S3,† all samples exhibit predominantly agglomerated particles with a similarly rough surface regardless of synthesis pH, which can be associated with the zirconia structure given its dominance in XRD results. As shown in Fig. 2A and B, such morphology is largely present in the pH10 sample, although here it also coexists with the characteristic rod-like ZnO particles^31^ shown in Fig. 2C. Fig. 2D illustrates the N_2_ sorption isotherms and pore size distributions of all the samples. All samples exhibit type-VI isotherms along with a type-H2 hysteresis loop. This characteristic behavior indicates the aggregation of ZrO_2_ particles, leading to the creation of interparticle voids within the materials.^32^ Elevating the pH synthesis values from 7 to 10 induced a noticeable shift in the position of the hysteresis loop towards higher relative pressures, suggesting a subtle enlargement in mesopore sizes within the material structure. Nevertheless, only limited changes in surface area are observed, as it gradually decreases from 70 m^2^ g^^−^1^ at pH 7 to 63 m^2^ g^−1^ at pH 10. As shown in Fig. 2E, all samples display narrow distributions of pore size, suggesting that a uniformity of pore sizes was achieved through the hydrothermal synthesis method.

**Fig. 3:**
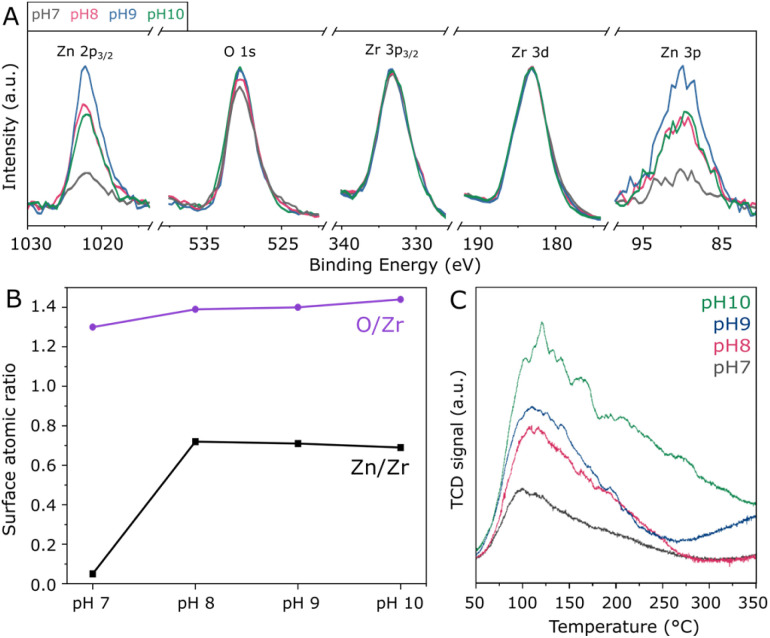
Survey XPS spectra of ZnZrO_*x*_ normalized by the Zr 3d peak, showing the Zn 2p_3/2_, O 1 s, Zr 3p_3/2_, Zr 3d and Zn 3p regions (A) with the respective quantification of the Zn/Zr and O/Zr surface molar ratios (B); CO_2_-TPD profiles of the samples produced at pH 7 to 10 (C).

In order to assess the surface composition of the investigated materials, XPS analysis was performed. According to high-resolution spectra in Fig. S4,† all samples present similar Zr 3d doublets with Zr 3d_5/2_ and Zr 3d_3/2_ in the region of 183.1 and 185.5 eV, corresponding to Zr^4+^ in tetragonal ZrO_2_.^33^ Accordingly, O 1s located at approximately 530.4 eV indicates that lattice oxygen^33^ is by far more abundant than adsorbed oxygen species.^34^ Moreover, the Zn 2p_3/2_ peak is verified at 1022.0 eV, as typically observed for Zn^2+^ species.^35^ Due to the severe differential charging^36^ experienced by the ZnZrO_*x*_ samples as a result of their insulating character and surface roughness, the tailing observed in the high-resolution XPS spectra prevents fitting or precise quantification in these spectra. For this reason, quantification of Zn/Zr and O/Zr surface atomic ratios was conducted in the survey spectra, considering O 1s, the average of Zn 2p_3/2_ and Zn 3p, as well as the average of Zr 3d and Zr 3p_3/2_ for higher precision. Accordingly, Fig. 3A presents these selected regions in the survey spectra, with the respective Zn/Zr and O/Zr surface molar ratios shown in Fig. 3B. Hydrothermal synthesis under neutral pH conditions results in a low surface Zn/Zr ratio of 0.05, which can be correlated with the observation of abundant monoclinic ZrO_2_ by XRD, as this phase is less likely to accommodate Zn^2+^.^16^ In contrast, the pH 8 sample shows a greatly increased Zn/Zr ratio of 0.72, which is slightly decreased to 0.71 and 0.69 upon increasing pH to 9 and 10, respectively. Despite the observed differences in crystal structure upon increasing synthesis pH from 8 to 10, these results suggest that the surface has a similar content of surface Zn^2+^ within this basic pH range. Also in Fig. 3B, the surface O/Zr ratio presents a steady increase from 1.30 to 1.44 between pH 7 and 10, giving an indication that hydrothermal conditions with abundant OH^−^ may provide more surface oxygen for the ZnZrO_*x*_ solid solution during calcination. Furthermore, even though Na^+^ from NaOH has been previously suggested to stabilize the t-ZrO_2_ structure, surface Na was not observed by XPS, as shown in Fig. S5.† However, the Cl 2p peak at 198 eV indicates surface chlorine species in all samples, as a residue from the metal chloride precursors utilized in the hydrothermal synthesis.^37^ Although the effect of such species has not been deeply explored in ZnZrO_*x*_ catalysts for CO_2_ hydrogenation to methanol, surface chlorine in Pd/ZnO was suggested to block some active sites and therefore offer a detrimental effect to catalytic activity in comparison to chlorine-free catalysts.^38^

Given the pivotal role of CO_2_ activation in its catalytic conversion, the CO_2_-TPD profiles of all ZnZrO_*x*_ samples are investigated, as shown in Fig. 3C. In all cases, a main desorption peak is observed below 200 °C, corresponding to the desorption of CO_2_ from weakly basic sites.^32^ Interestingly, the material produced at pH 7 presents the lowest CO_2_ adsorption capacity despite having the highest surface area among the investigated samples. This observation cannot be directly associated with the presence of m-ZrO_2_, as this phase typically interacts more strongly with CO_2_ than t-ZrO_2_.^39^ As the synthesis pH rises from 7 to 10, this desorption peak position becomes more intense and is gradually shifted from 100 to 120 °C, indicating an increase in its basicity. Concurrently, higher synthesis pH values lead to increased desorption between 200 and 350 °C, indicating higher densities of moderately basic sites. This can be considered more relevant for CO_2_ hydrogenation to methanol,^21^ as this reaction is typically conducted around 250 °C. Such positive correlation between synthesis pH and CO_2_ adsorption capacity could be connected with the formation of the ZnZrO_*x*_ solid solution, which may show higher CO_2_ adsorption with respect to pure ZrO_2_.^40^

**Fig. 4:**
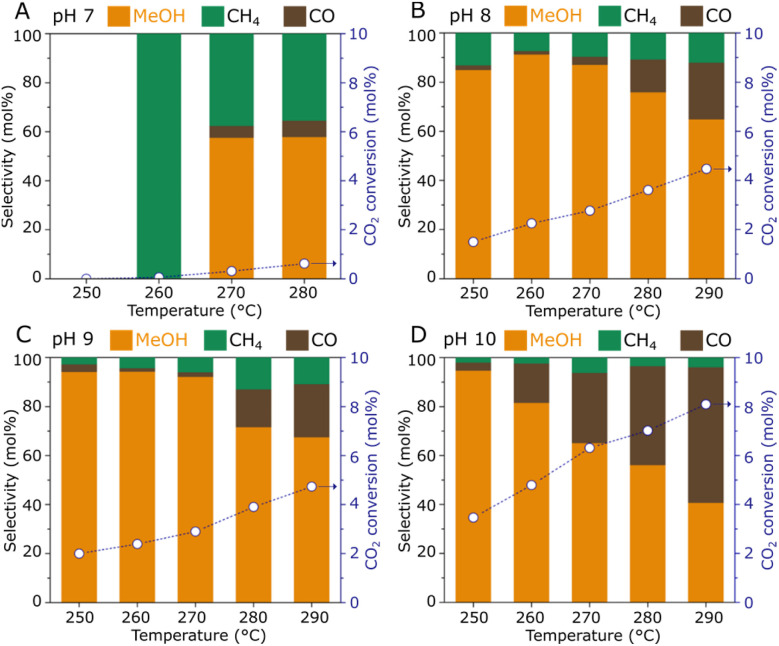
CO_2_ hydrogenation performance of ZnZrO_*x*_ catalysts produced under synthesis pH 7 (A), 8 (B), 9 (C), and 10 (D). Reaction conditions: 0.5 g catalyst, 2.0 MPa, CO_2_/H_2_ = 1/3, 5 mL min^−1^.

The catalytic hydrogenation of CO_2_ to methanol over the ZnZrO_*x*_ samples was tested in a fixed-bed reactor at various temperatures (250–290 °C), as summarized in Fig. 4. The material produced under pH 7 exhibits poor catalytic activity with CO_2_ conversion below 1%, as a possible result of the low surface Zn content observed by XPS, since the Zn^2+^–O–Zr^4+^ linkages have been widely recognized as the active sites in ZnZrO_*x*_ catalysts. However, the pH 8 sample shows a sharply enhanced catalytic activity over the entire temperature range, as a likely effect of the improved incorporation of Zn^2+^ in the tetragonal zirconia phase. In this case, a CO_2_ conversion of 1.5% and a methanol selectivity of 84% are achieved at 250 °C, and increasing the reaction temperature to 290 °C results in a CO_2_ conversion of 4.5% and a methanol selectivity of 65%, as higher temperatures simultaneously favor the kinetics for CO_2_ hydrogenation to methanol and the endothermic production of CO *via* Reverse Water-Gas Shift reaction. Interestingly, increasing synthesis pH to 9 and 10 leads to progressively higher CO_2_ conversions. As shown in Fig. 4D, at the highest pH value a CO_2_ conversion of 3.4% is obtained along with a methanol selectivity of 95% at 250 °C. Such value is in a similar range to the previously reported performance at 260 °C/2.0 MPa using ZnZrO_*x*_ produced by co-precipitation.^11^ Accordingly, an expressive increase in CO_2_ conversion to 8.1% is observed at 290 °C, although this coincides with a decreased methanol selectivity of 40%.

In view of the catalytic performance of ZnZrO_*x*_ produced *via* hydrothermal synthesis at distinct pH values between 7 and 10, a clearly positive correlation between synthesis pH and CO_2_ conversion is verified. As pH values increase, catalytic activity at 250 °C is enhanced with high methanol selectivity, while at higher temperatures this also coexists with an enhanced production of CO. At pH 10, the higher CO production above 250 °C could be associated with the additional contribution of ZnO, similarly as previously observed when ZnZrO_*x*_ is produced with an exceedingly high Zn content.^11^ Although the formation of abundant Zn^2+^/t-ZrO_2_ sites is important in this catalyst, surface characterization suggests that in this study, catalytic activity cannot be simply associated with surface area and the Zn surface content, as these parameters do not increase between pH 8 and 10. This is consistent with the observation that H_2_ activation may not simply require Zn^2+^ atoms but rather Zn^2+^–O^2−^ pairs, as suggested by previous studies.^19,20^ Therefore, the enhanced CO_2_ conversion to methanol may be related to the stronger CO_2_ adsorption capacity^21^ and with the slightly higher lattice oxygen content at the catalyst surface, which may in turn benefit H_2_ dissociation.^19,20^ Despite the correlation of synthesis pH with catalytic activity for CO_2_ hydrogenation to methanol, the observed trends indicate that further increasing pH beyond 10 could lead to a ZnZrO_*X*_/ZnO system with lower selectivity due to the production of CO as a byproduct.

**Fig. 5:**
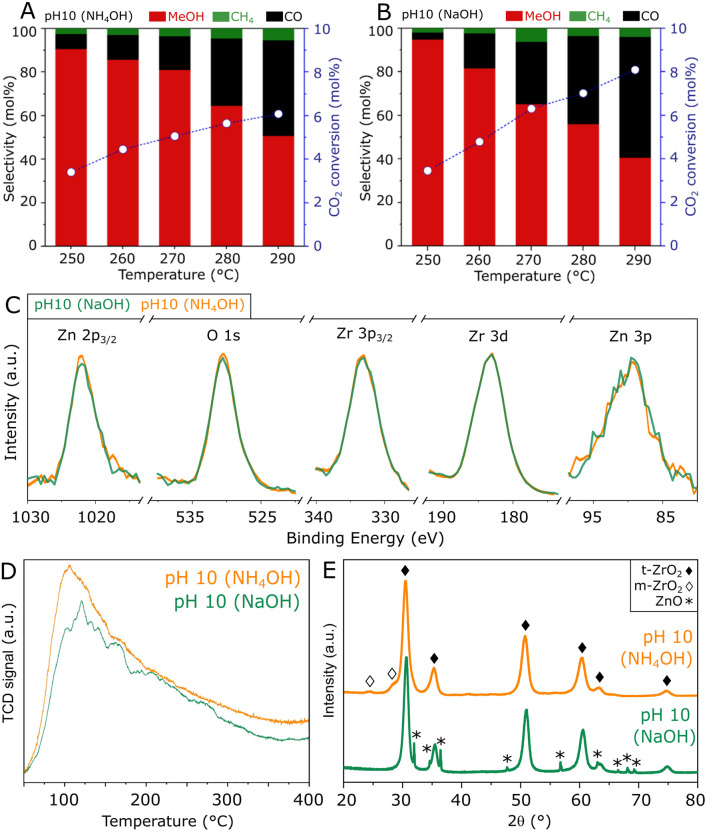
Comparison of ZnZrO_*x*_ catalysts produced under synthesis pH 10 with the distinct additives NH_4_OH and NaOH, in terms of CO_2_ hydrogenation performance (A and B), Zn 2p_3/2_, O 1s, Zr 3p_3/2_, Zr 3d and Zn 3p XPS spectra (C), CO_2_-TPD profiles (D) and XRD patterns (E).

To obtain further insights into the hydrothermal synthesis of ZnZrO_*x*_ catalysts, an analogous preparation procedure was followed using NH_4_OH to achieve pH 10, as an alternative to NaOH. A comparison of such materials, shown in Fig. 5A and B, shows similar catalytic activities at 250 °C, as the catalyst produced with NH_4_OH presents an unchanged CO_2_ conversion of 3.4% with a slightly lower methanol selectivity of 90%. At reaction temperatures higher than 250 °C, the material shows similar methanol production but improved selectivity due to the lower production of CO. Corresponding to such similarities, ZnZrO_*x*_ produced at pH 10 with NH_4_OH and NaOH show similarly high O/Zr ratios of 1.47 and 1.44, calculated from the XPS spectra in Fig. 5C. Furthermore, the comparable CO_2_-TPD profiles in Fig. 5D indicate a similarly high density of weakly and moderately basic sites, with respect to ZnZrO_*x*_ produced at lower pH. Interestingly, the XRD patterns in Fig. 5E indicate that the main contribution consists of t-ZrO_2_ in both catalysts, but when NH_4_OH is used, m-ZrO_2_ appears as a minor phase instead of ZnO. Given the most likely negative effect of ZnO, such differences in crystallinity suggest that the absence of ZnO may explain the higher methanol selectivity above 250 °C in the catalyst produced with NH_4_OH. Although this finding hints at a possible improvement by further increasing synthesis pH using NH_4_OH, reaching values above 10 was not feasible without drastically altering the content of the remaining reactants involved in the hydrothermal synthesis of ZnZrO_*x*_.

In summary, these results emphasize that high surface oxygen content and basicity for CO_2_ activation are key features behind the high catalytic activity of ZnZrO_*x*_ produced *via* hydrothermal synthesis at pH 10. As expected, these findings confirm that the Zn^2+^–ZrO_2_ solid solution is the active phase in the catalyst, and the presence of bulk ZnO may not contribute to methanol production in this case, even though the presence of ZnO_*x*_ clusters within the solid solution cannot be ruled out.^17,18^ In fact, bulk ZnO is shown to have a detrimental effect on methanol selectivity as it promotes the formation of CO as a byproduct.

**Fig. 6:**
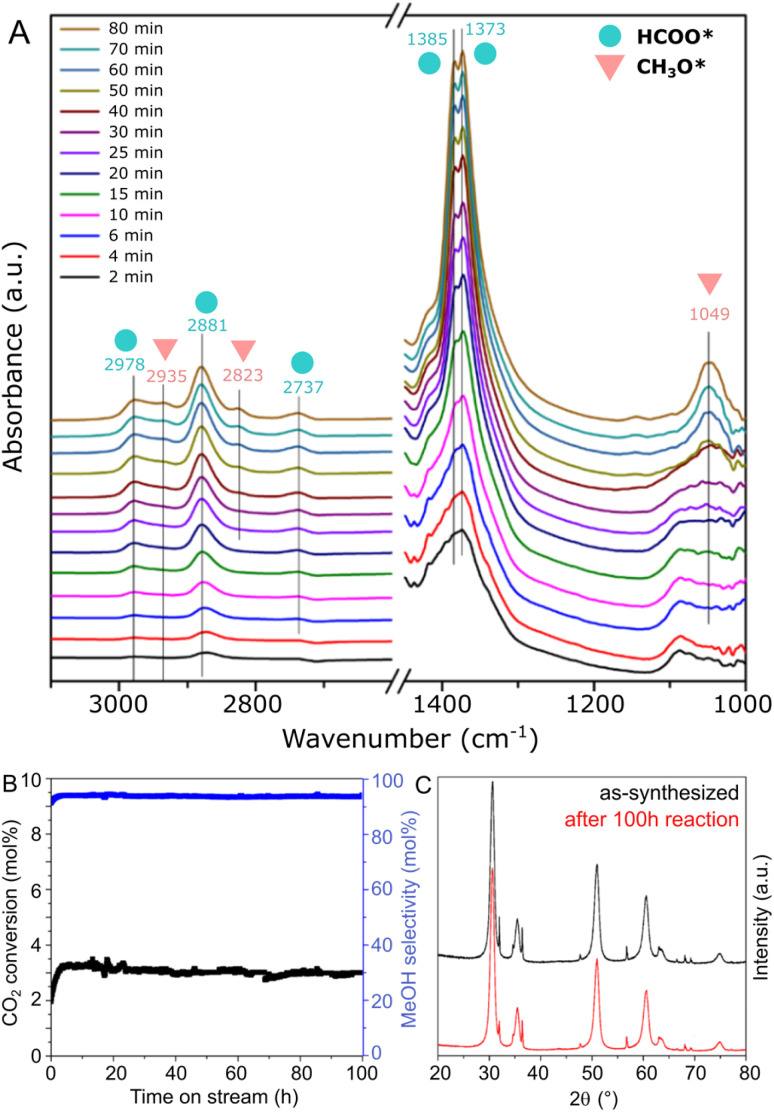
*In situ* DRIFTS results of CO_2_ hydrogenation conditions at 250 °C (A) and catalytic stability test at 250 °C using the ZnZrO_*x*_ catalyst produced at pH 10 with NaOH (B), with the respective XRD patterns of as synthesized and spent material (C).

In order to obtain insights on the reaction mechanism of CO_2_ hydrogenation to methanol over the ZnZrO_*x*_ catalyst produced at pH 10, the surface intermediates involved in the reaction were monitored by an *in situ* DRIFTS experiment. Spectra were collected at 250 °C under CO_2_/H_2_ flow at ambient pressure, as shown in Fig. 6A. Specifically, the peaks observed at 2978, 2881, 2737, 1385, and 1373 cm^−1^, which appear in the initial 2 minutes of the experiment, can be ascribed to formate species (HCOO*). Subsequently, after approximately 25 min, additional peaks corresponding to CH_3_O* are observed at 2935, 2823, and 1049 cm^−1^, with progressively increased intensity over the reaction time. These results suggest that CH_3_O* species are generated through the stepwise hydrogenation of HCOO* species, as part of a formate reaction pathway previously suggested in other ZnZrO_*x*_ catalysts.^11,22,41^

In Fig. 6B, the stability of the ZnZrO_*x*_ catalyst is evaluated through a 250 °C reaction carried out during 100 h. The material demonstrates remarkable stability over the test period, with no deactivation trend observed in both CO_2_ conversion and methanol selectivity over the 100 hours duration. Correspondingly, Fig. 6C shows that the XRD patterns of spent and fresh catalyst are highly similar, apart from a minor decrease in the peak intensities of ZnO. This change might be attributed to a slight amorphization of ZnO facilitated by the reducing reaction conditions. Nevertheless, given the key role of the Zn^2+^/t-ZrO_2_ solid solution for CO_2_ hydrogenation to methanol, these patterns suggest high catalyst robustness under reaction conditions.

## Conclusions

In the hydrothermal synthesis of ZnZrO_*x*_ catalysts for CO_2_ hydrogenation to methanol, altering synthesis pH significantly impacts both the structural and catalytic properties of the material. Basic pH conditions promote the formation of tetragonal ZrO_2_ and facilitate the incorporation of Zn^2+^ into this phase. As synthesis pH is increased from 8 to 10, the catalysts show a marked improvement in methanol production at 250 °C, while higher temperatures favor CO production. The correlation between hydrothermal synthesis pH and catalytic activity may be associated with the improved surface basicity verified by CO_2_-TPD and the slight increase in the surface oxygen content observed by XPS, given the decisive effect of Zn^2+^–O^2−^ pairs on H_2_ activation.^20^ At reaction temperatures above 250 °C, the selectivity of CO_2_ hydrogenation to methanol can be improved by utilizing NH_4_OH as an alternative to NaOH in the hydrothermal synthesis at pH 10, thus suppressing the formation of bulk ZnO. In summary, these findings suggest that the hydrothermal approach is an effective and versatile method for producing ZnZrO_*x*_ catalysts for CO_2_ hydrogenation to methanol. Nevertheless, given the inherently limited hydrogen activation in ZnZrO_*x*_, achieving catalytic activity superior to commercial Cu-based catalysts will likely require further strategies, such as the optimization of Zn^2+^ dispersion^42^ or the addition of metallic nanoparticles^13,43^ and promoters,^22^ which could offer new concepts for the hydrothermal synthesis of ZnZrO_*x*_-based catalysts.

## Data availability

The data supporting this article have been included as part of the ESI.†

## Conflicts of interest

There are no conflicts to declare.

## Supplementary Material

SU-002-D4SU00522H-s001
