# Correction: Hydrothermal synthesis of ZnZrO_*x*_ catalysts for CO_2_ hydrogenation to methanol: the effect of pH on structure and activity

**DOI:** 10.1039/d4su90059f

**Published:** 2024-11-11

**Authors:** Issaraporn Rakngam, Gustavo A. S. Alves, Nattawut Osakoo, Jatuporn Wittayakun, Thomas Konegger, Karin Föttinger

**Affiliations:** a School of Chemistry, Institute of Science, Suranaree University of Technology Nakhon Ratchasima 30000 Thailand; b Institute of Materials Chemistry, TU Wien Getreidemarkt 9 1060 Vienna Austria karin.foettinger@tuwien.ac.at; c Institute of Research and Development, Suranaree University of Technology Thailand; d Institute of Chemical Technologies and Analytics, TU Wien Getreidemarkt 9 1060 Vienna Austria

## Abstract

Correction for ‘Hydrothermal synthesis of ZnZrO_*x*_ catalysts for CO_2_ hydrogenation to methanol: the effect of pH on structure and activity’ by Issaraporn Rakngam *et al.*, *RSC Sustain.*, 2024, https://doi.org/10.1039/d4su00522h.

The authors regret that, in the original article, there was an error in the affiliation details for Gustavo Alves and Karin Föttinger, whereby a redundant affiliation was associated with both authors. The corrected author affiliation list, with the redundant entry removed and the remaining entries relettered accordingly, is provided in this document.

The Royal Society of Chemistry apologises for these errors and any consequent inconvenience to authors and readers.

