# Supplementary material for: Hydrothermal synthesis of ZnZrOx catalysts for CO2 hydrogenation to methanol: the effect of pH on structure and activity
Source: RSC Sustain. 2024 Oct 11;2(12):3798–805. doi: 10.1039/d4su00522h (PMC11484158; doi:10.1039/d4su00522h)
Supplement: SU-002-D4SU00522H-s001 [file SU-002-D4SU00522H-s001.pdf]

Supplementary Information (SI) for RSC Sustainability.
This journal is © The Royal Society of Chemistry 2024

Hydrothermal synthesis of $ZnZrO_x$ catalysts for CO_2 hydrogenation to methanol: the effect of pH on structure and activity

Issaraporn Rakngam[a]*, Gustavo A. S. Alves[b]*, Nattawut Osakoo[c], Jatuporn Wittayakun[a], Thomas Konegger[d], Karin Föttinger[b,†]

*These authors have contributed equally to this work

† Corresponding Author

[a]School of Chemistry, Institute of Science, Suranaree University of Technology, Nakhon Ratchasima, 30000, Thailand

[b]Institute of Materials Chemistry, TU Wien, Getreidemarkt 9, 1060, Vienna, Austria

[c]Institute of Research and Development, Suranaree University of Technology, Thailand

[d]Institute of Chemical Technologies and Analytics, TU Wien, Getreidemarkt 9, 1060, Vienna, Austria

Supporting Information

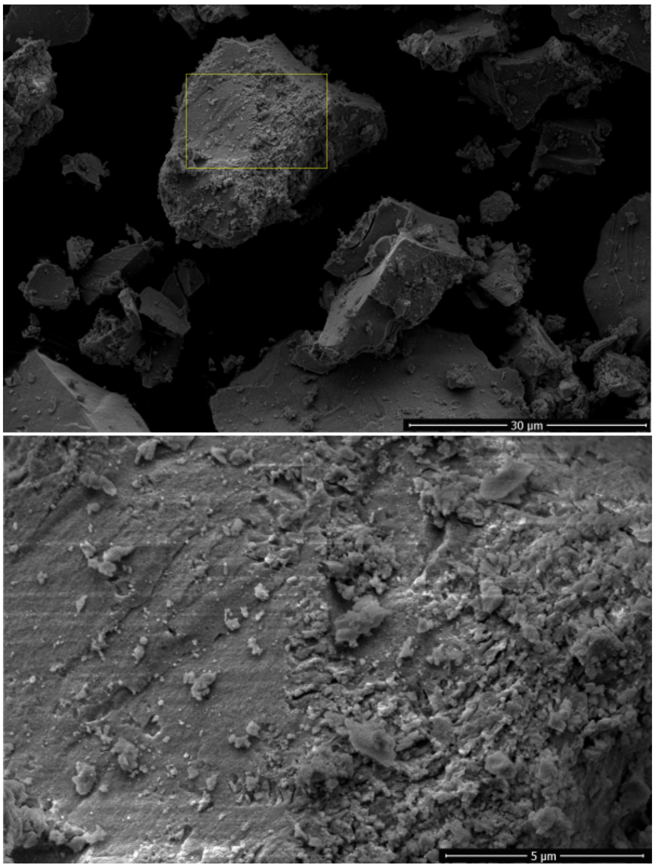

Figure S1 - SEM micrographs of ZnZrOx produced under pH 7

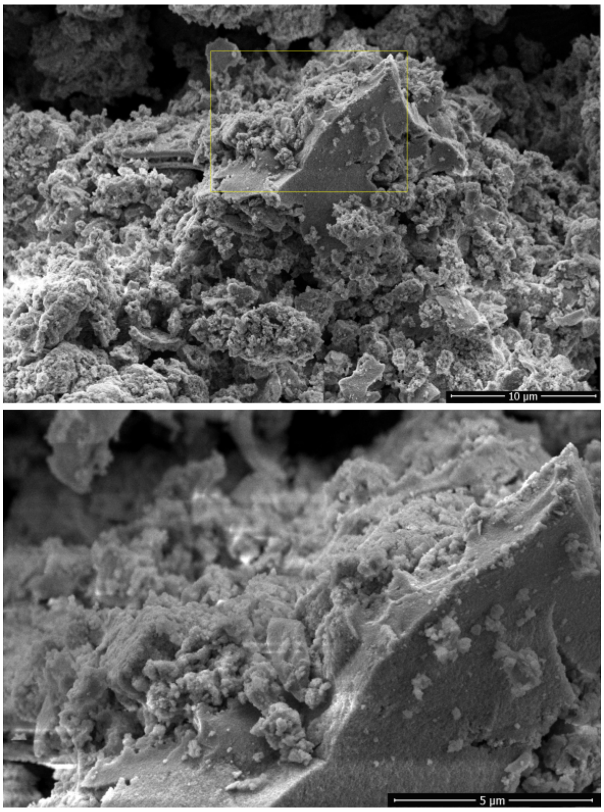

Figure S2 - SEM micrographs of ZnZrOx produced under pH 8

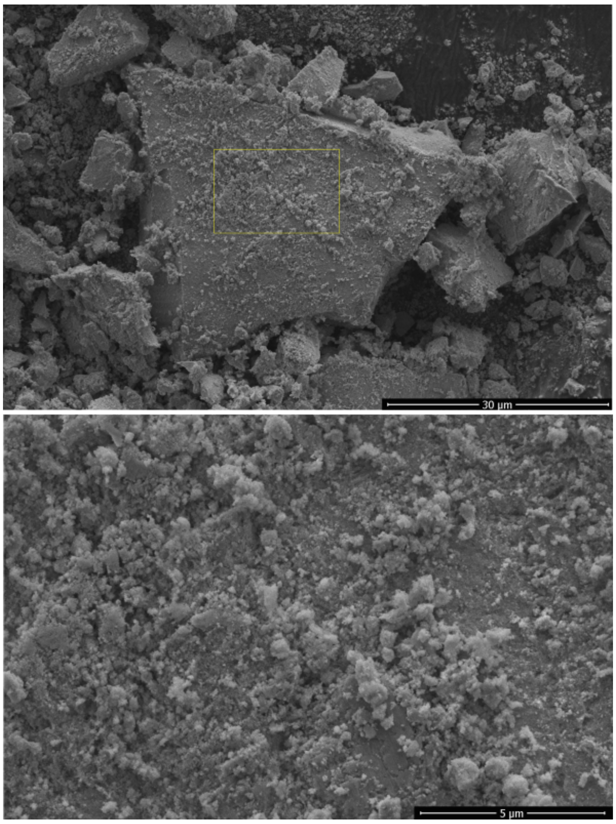

Figure S3 - SEM micrographs of ZnZrOx produced under pH 9

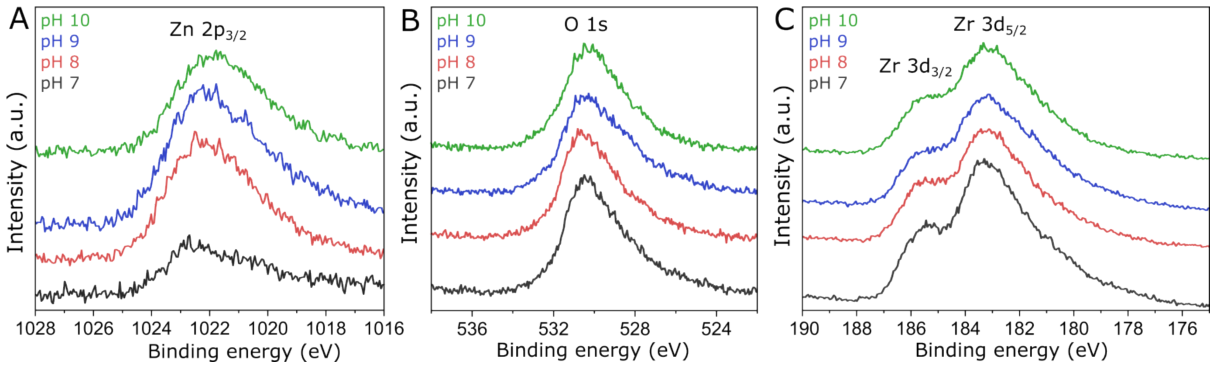

Figure S4 - High-resolution XPS spectra of samples produced at pH 7-10 using NaOH, showing Zn 2p (A), O 1s (B) and Zr 3d (C) regions.

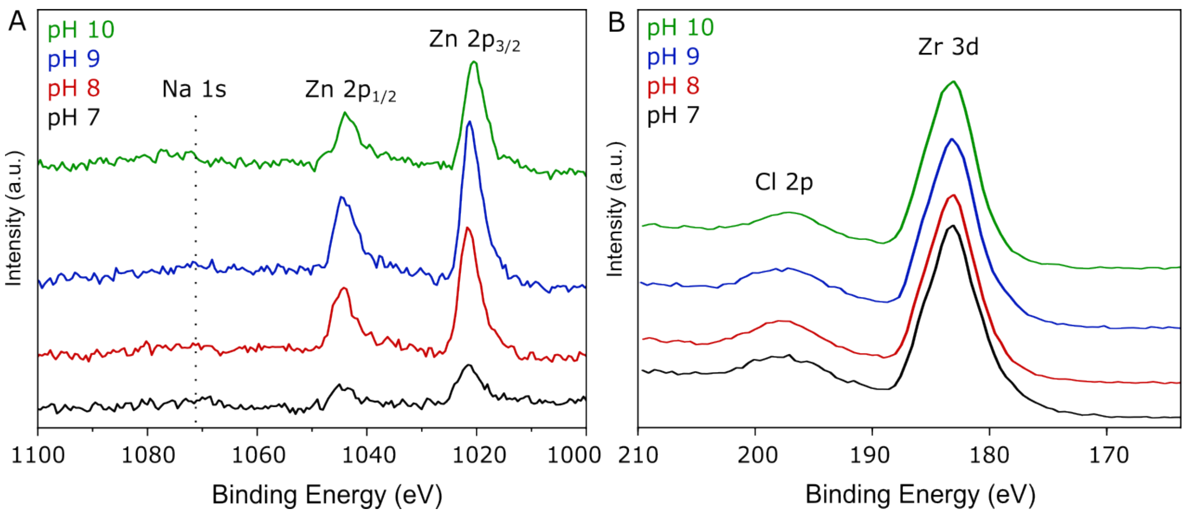

Figure S5 - Survey XPS spectra of samples produced under pH 7, 8, 9 and 10, highlighting the absence of the Na 1s peak (A) and the presence of the Cl 2p peak (B)

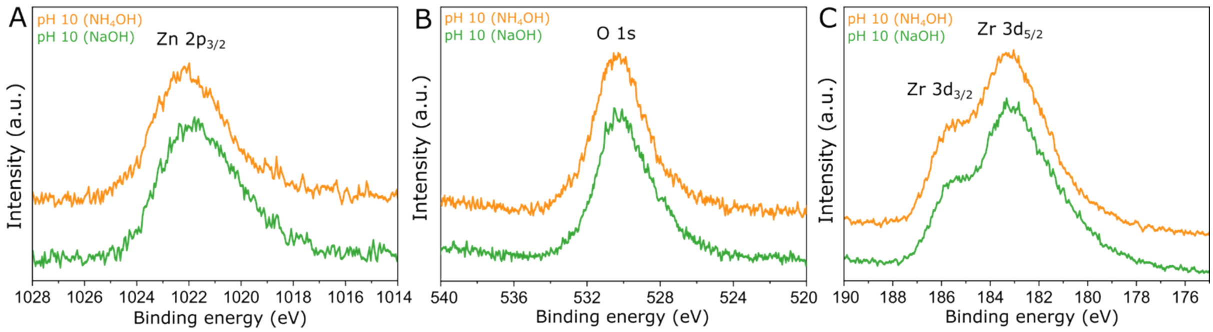

Figure S6 - High-resolution XPS spectra of samples produced at pH 10 with NaOH and NH_4OH, showing Zn 2p (A), O 1s (B) and Zr 3d (C) regions.

Table S1 - Surface properties of ZnZr produced with different synthesis pH values.

Sample	Textural properties (BET)		Zn/Zr from XPS	O/Zr from XPS
	Surface area (m^2/g)	Pore size (nm)		
ZnZrOx pH7	70	4.3	0.05	1.30
ZnZrOx pH8	67	4.8	0.72	1.39
ZnZrOx pH9	65	6.8	0.71	1.40
ZnZrOx pH10	63	10.5	0.69	1.44
